# 7‐Step Flow Synthesis of the HIV Integrase Inhibitor Dolutegravir

**DOI:** 10.1002/anie.201802256

**Published:** 2018-05-14

**Authors:** Robert E. Ziegler, Bimbisar K. Desai, Jo‐Ann Jee, B. Frank Gupton, Thomas D. Roper, Timothy F. Jamison

**Affiliations:** ^1^ Department of Chemistry Massachusetts Institute of Technology 77 Massachusetts Avenue Cambridge MA 02139 USA; ^2^ Department of Chemical and Life Science Engineering Virginia Commonwealth University, Biotech 8 737 N. 5^th^ Street Richmond VA 23219 USA

**Keywords:** amidation, continuous flow, HIV, multistep synthesis, pyridone

## Abstract

Dolutegravir (DTG), an important active pharmaceutical ingredient (API) used in combination therapy for the treatment of HIV, has been synthesized in continuous flow. By adapting the reported GlaxoSmithKline process chemistry batch route for Cabotegravir, DTG was produced in 4.5 h in sequential flow operations from commercially available materials. Key features of the synthesis include rapid manufacturing time for pyridone formation, one‐step direct amidation of a functionalized pyridone, and telescoping of multiple steps to avoid isolation of intermediates and enable for greater throughput.

HIV is a disease that currently affects around 37 million people.^1^ A number of innovative medicines has made HIV a manageable disease; however, the cost of treatment is still prohibitive for many patients in lower income countries.^2^ As part of the “Medicines for All” initiative funded by the Bill and Melinda Gates Foundation,^3^ our research groups are focused on providing greater access to essential medicines for serious diseases such as HIV, tuberculosis, and malaria. The initiative has led to a number of reported studies.^4^ We became interested in Dolutegravir due to its importance in HIV combination therapy.

Dolutegravir (DTG) **1** (Figure 1) is an HIV integrase inhibitor co‐developed by GlaxoSmithKline (GSK) and Shinogi that was approved by the Food and Drug Administration (FDA) in 2013. Integrase inhibitors prevent the HIV virus from inserting into cellular DNA by blocking transesterification, a process that is vital for replication and spread of the disease.^5^ Raltegravir **2** and Elvitegravir **3** were the first integrase inhibitors to be approved and used in combination therapy; however, these two drugs require large doses and a pharmacokinetic booster, respectively, and have shown vulnerability to HIV virus mutations.^6^ DTG is an un‐boosted, once daily 50 mg tablet that is recommended as a universal first‐line treatment in combination therapy due to its low dosage and limited side effects.^7^ Only minimal resistance has been observed thus far, which has not led to significant spread of HIV virus after mutation.^6^ The high resistance and minimal side effects have led DTG to be placed on the World Health Organization's List of Essential Medicines.^8^ Recently, the first two drug combination therapy for HIV (Dolutegravir and Rilpivirine) was approved by the FDA.^9^ A number of DTG analogues are currently in clinical trials, including Cabotegravir **4** and Bictegravir **5**; the latter was recently approved by the FDA in a single tablet, three drug regimen (Figure 1).^10^ These analogues differ from Dolutegravir **1** in the oxazine ring size; thus, a synthesis for DTG should also be amenable to **4** or **5** if they emerge as the integrase inhibitor of choice in the future.

**Figure 1:**
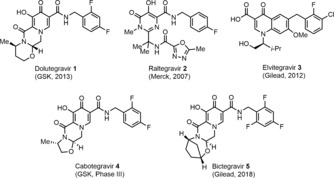
Integrase inhibitors for HIV treatment.

GSK and Shinogi disclosed a number of approaches to the synthesis of **1** and its analogues.^11^ Initial medicinal chemistry routes exploited commercially available heterocycles such as nicotinic acid and maltol, and subsequently installed the requisite functionalized N‐H pyridone in ten or more steps.11b–11d Wang and co‐workers from GSK later published a highly efficient, chromatography‐free approach to Cabotegravir **4** through rapid formation of the functionalized pyridone core **8** and subsequent cyclization with (*S*)‐alaninol **11** to synthesize the 5‐membered oxazine ring (Scheme 1).11e,11f We wished to optimize and adapt the synthesis to a continuous flow system in order to streamline manufacturing of the API.^12^ Continuous flow reactions benefit from increased mixing due to high surface area to volume ratio and the ability to heat solvents well past their boiling point.^13^ By developing an efficient flow synthesis and telescoping steps to avoid purifications, we felt we could achieve a short, scalable synthesis of DTG **1**.

**Scheme 1:**
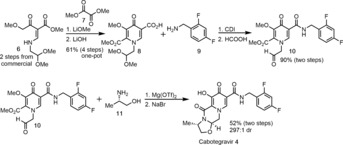
GSK process synthesis of Cabotegravir **4**.

We began our investigation with the condensation reaction of methyl 4‐methoxyacetoacetate **12** and dimethylformamide dimethylacetal (DMF‐DMA) **13**. Our first attempt at the analogous flow reaction proceeded with 60 % conversion to **14** when an equimolar ratio of the neat reactants **12** and **13** were streamed through a T‐mixer at 30 °C with a residence time (*t*_R_) of 10 min. Further optimization led to the discovery that an elevated reaction temperature of 85 °C and 1.6 equiv of DMF‐DMA **13** resulted in full conversion of **12** to **14** via HPLC with the same 10 min residence time.^14^ With an efficient approach to the dimethyl vinylogous amide **14**, we next sought to telescope Steps 1 and 2 in continuous flow (Scheme 2). This was achieved by connecting Reactor I with a T‐mixer and adding neat aminoacetaldehyde dimethylacetal **15** directly to the output of Reactor I. Due to the high crystallinity of the product **6**, we conducted Step 2 at 85 °C to increase solubility and avoid clogging. The optimized telescoped flow process produced **6** in an isolated yield of 95 % and a throughput of 43 g h^−1^.

**Scheme 2:**
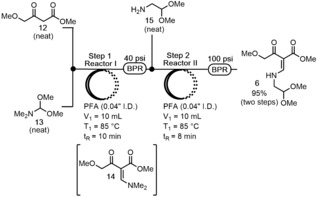
Telescoped flow synthesis of vinylogous amide **6**. PFA=perfluoroalkoxy, I.D.=inside diameter.

The next step in the reaction sequence was the pyridone **16** formation (Scheme 3). Given the complexity of adapting to continuous flow, we first examined the independent flow process by starting with purified vinylogous amide **6**. The choice of solvent for the reaction proved to be crucial given both **6** and dimethyl oxalate **7** are solids. A solvent screen in batch showed that CH_3_CN afforded the highest conversion (93 %) to product **16** compared to *N*‐methylpyrrolidinone and MeOH (both 85 %); however the CH_3_CN condition suffered from poor solubility that led to clogging in flow.^14^ In addition, when examining different bases for the deprotonation/cyclization sequence, NaOMe in MeOH had much better solubility and conversion to **16** than LiOMe, which was utilized in the GSK synthesis.11e,11f These screening results in batch led us to investigate Step 3 in flow using NaOMe as base and MeOH as solvent in order to simplify the system through the use of a single solvent. Following optimization of residence time and temperature,^14^ flow conditions of 85 °C and 30 min *t*_R_ led to a 91 % isolated yield of **16** (Scheme 3).

**Scheme 3:**
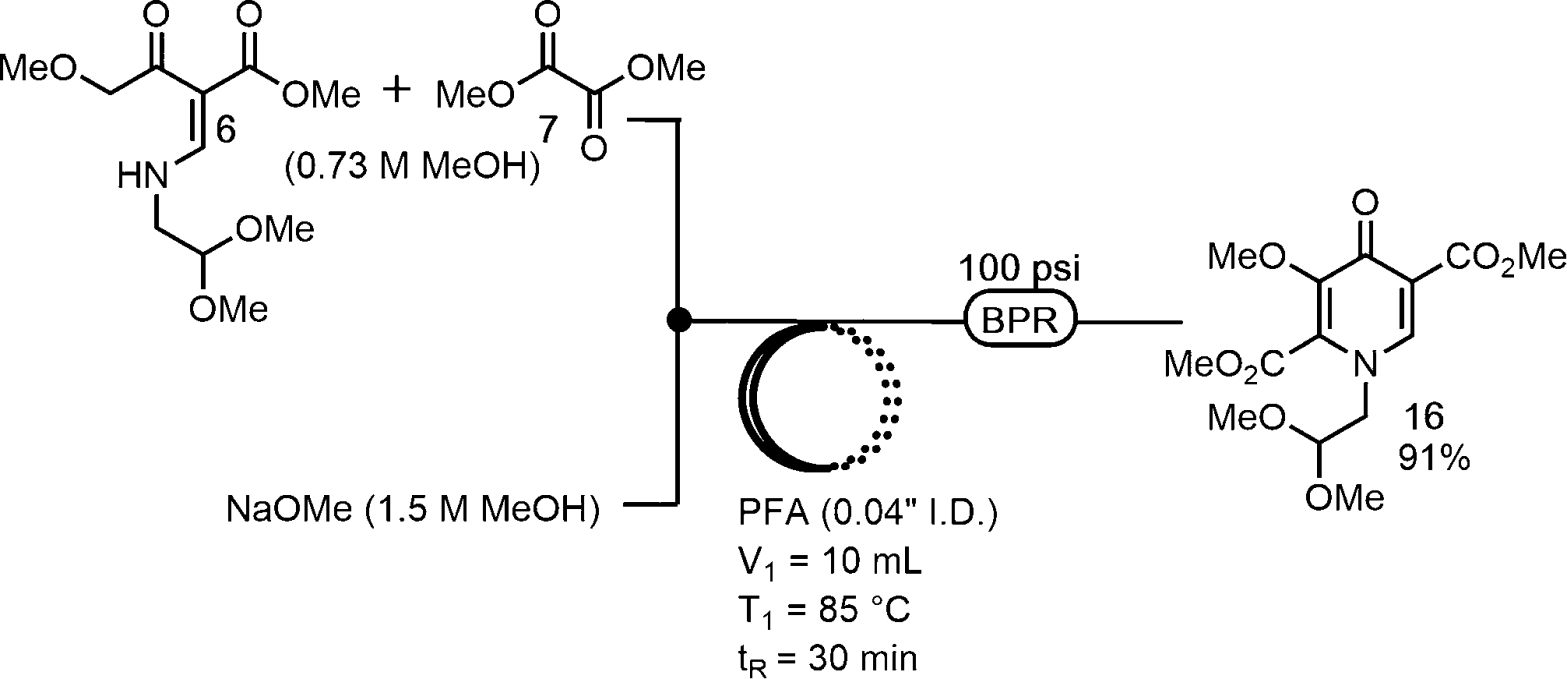
Pyridone **16** formation in flow.

Next, we examined the synthesis of pyridone **16** from **12** in a three‐step telescope process to obviate time consuming purification operations and work towards our goal of a fully continuous synthesis (Scheme 4). After minor modifications to the previously optimized flow conditions from Scheme 2 and Scheme 3, the telescoped synthesis of **16** was achieved. In this setup, several 40 psi back pressure regulators (BPR) were utilized as check valves and Reactor III was used to facilitate premixing of solution **7** with the output from Reactor II prior to the addition of NaOMe. A 55 min residence time in Reactor IV was required for the reaction to reach completion. The three‐step telescoped synthesis of pyridone **16** led to a 56 % isolated yield in a total residence time of 74 min with a throughput of 3.4 g h^−1^.

**Scheme 4:**
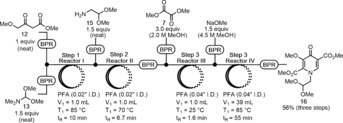
Three‐step telescoped synthesis of pyridone **16**.

When considering ways to shorten the reported GSK synthesis, the ester saponification to form **8** and subsequent amide coupling stood out as an opportunity. Specifically, in GSK's synthesis of Cabotegravir **4** (Scheme 1),11e,11f ester **16** was saponified to the 5‐carboxylic acid **8** and then treated with carbonyldiimidazole (CDI) and difluorobenzylamine **9** to form amide product **17**. The process required a filtration and extraction, and took 17 h in total. We were encouraged by a similar direct amidation that was reported in the midst of our own studies.^15^ Application of Kumar and co‐workers’ AcOH‐catalyzed conditions led to high yield and chemoselectivity for amidation at the 5‐position, producing amide **17** after 10 h (Scheme 5 a). We reason that there is both an electronic and steric effect which led to the selectivity. When the corresponding N‐H pyridone was reacted under the same conditions, a 2:1 selectivity for 5‐amidation over 2‐amidation was observed. The only byproducts observed on a gram scale batch reaction of **16** were ring opened starting material **6** and the analogous difluorobenzyl vinylogous amide (<5 % each). An ethyl ester substrate gave comparable yield and AcOH was found to be necessary for practical reaction time.^16^ A brief screen of Brønsted and Lewis acids revealed that the batch reaction could be accelerated compared to the AcOH condition, but with a decrease in isolated yield due to other byproducts and generally poorer solubility.

**Scheme 5:**
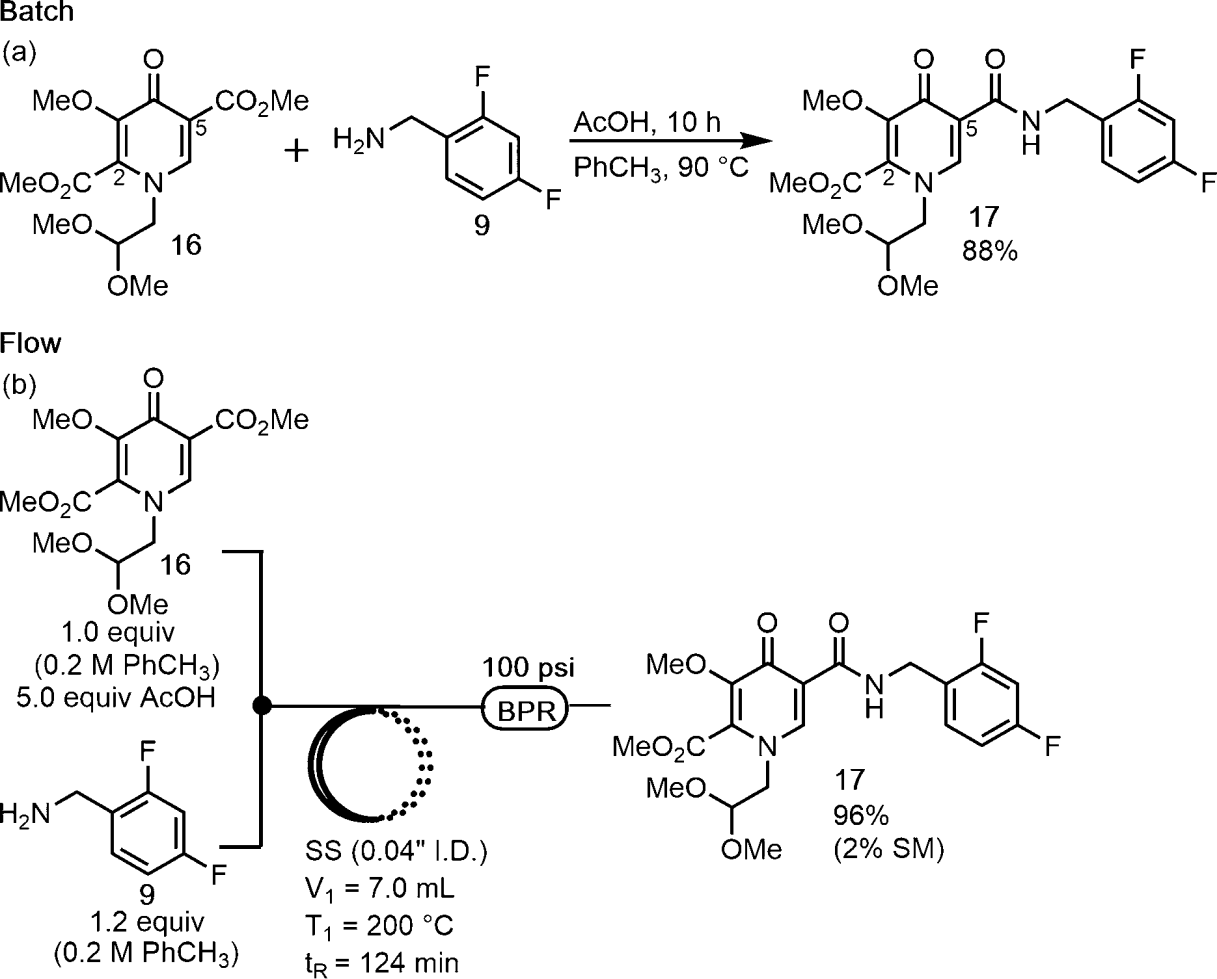
Direct amidation comparison. SS=stainless steel.

The next step was to adapt the process to a flow setup. Initially, we examined the reaction at 120 °C with a residence time of 1 h. Gratifyingly, product **17** was obtained with the use of a 100 psi BPR albeit in low conversion of starting material **16**. A systematic evaluation of temperature and residence time led to good conversion at 150 °C with a 1 h residence time and at 180 °C with a 30 min residence time.^14^ We screened other solvents and found that dioxane and CH_3_CN were viable, but gave lower conversion than PhCH_3_ in the flow setup. DCE led to clogging in the system, and DMF and MeOH failed to produce any desired amide **17** after 1 h. Under the optimal conditions of 200 °C with a residence time of 124 min, we obtained 96 % isolated yield of amide **17** (2 % recovered **16**) on a 3 mmol scale, which amounted to 3.5 g h^−1^ (Scheme 5 b).

The analogous base‐promoted amidation^17^ was also feasible using either LiOMe or NaOMe as base in a MeOH/PhCH_3_ mixed solvent system. The optimized flow process led to both shorter residence time and milder temperature compared to the acid‐mediated method (Scheme 6). However, attempts to telescope the basic amidation into the subsequent downstream process led to extensive clogging issues.

**Scheme 6:**
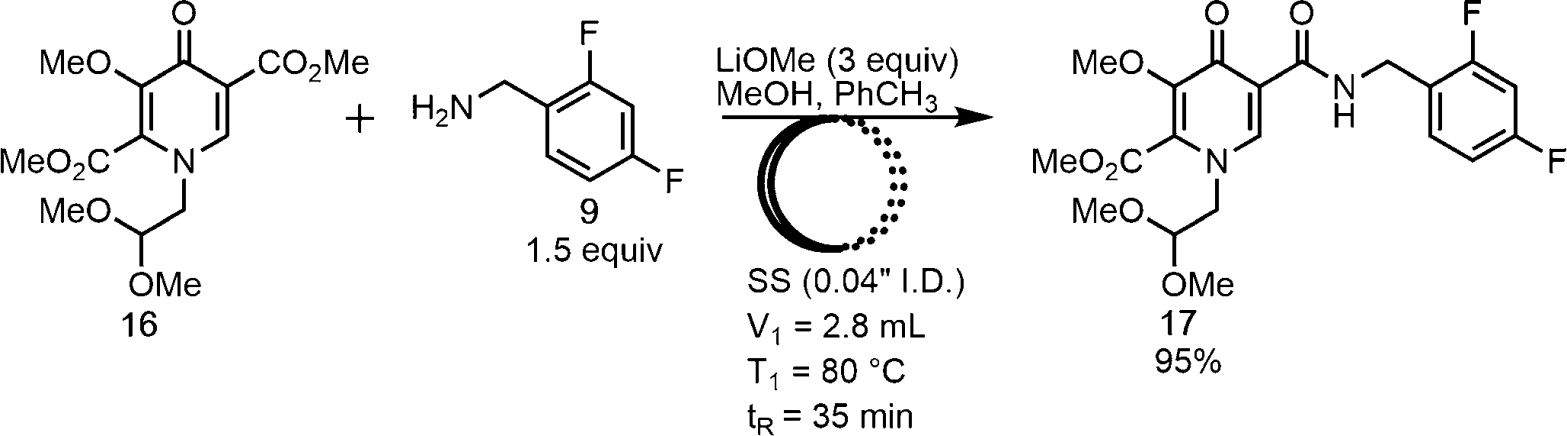
Base‐promoted direct amidation in flow.

Next, we examined the acetal deprotection of **17** and cyclization with (*R*)‐3‐aminobutan‐1‐ol **18** in flow. Initial attempts were conducted on purified pyridone amide **17** with an acid additive and amino alcohol **18**. Ultimately, we found that formic acid was required as a co‐solvent to observe any conversion of **17**. Our best result afforded an 8 % yield of DTG‐OMe **19** as a 5:1 mix of diastereomers favoring the desired product (Scheme 7).^18^ The major byproducts were ring‐opened **20**, produced through elimination of the hemiaminal ether functionality, and the deprotected pyridone aldehyde **10**. We reasoned that a two‐step flow procedure in which an acid would deprotect acetal **17** in one reactor and then meet amino alcohol **18** in a subsequent reactor may be more fruitful for conversion and milder conditions. Gratifyingly, separating the steps gave full conversion and allowed for stoichiometric amounts of *p*‐TsOH⋅H_2_O instead of neat formic acid with no observation of elimination byproduct **20**.

**Scheme 7:**
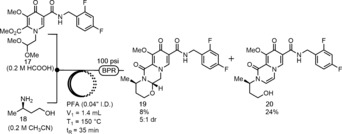
Initial cyclization attempts.

We next sought to telescope the acid‐mediated direct amidation into the deprotection/cyclization steps to form a three‐step telescoped sequence. Initially, incomplete conversion was observed due to issues with PhCH_3_ from Step 4 inhibiting the subsequent steps; however, it was eventually found that more concentrated reaction solutions and longer residence times in Steps 5 and 6 led to full conversion of intermediate amide **17** and aldehyde **10**. The optimized setup proceeded in a total residence time of just over 3 h and a 48 % isolated yield of DTG‐OMe **19** in 7:1 dr (Scheme 8). The major diastereomer was separated by silica gel chromatography to give analytically pure material.

**Scheme 8:**
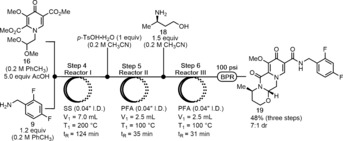
Telescoped synthesis of DTG‐OMe **19**.

Finally, we examined the demethylation step in continuous flow as a discrete operation using purified **19**. Following a batch screen of different demethylating reagents, it was found that GSK's published reaction using LiBr gave the best conversion and lowest amount of byproducts.11e,11f Reaction temperatures higher than 120 °C led to formation of a similar elimination byproduct to **20**. The batch conditions translated well to flow, with 89 % yield of DTG observed at 100 °C with a residence time of 31 min (Scheme 9). The reaction concentration proved crucial to reproducible, extended running of the continuous flow reactor. DTG **1** was insoluble upon cooling to room temperature, which led to clogging at concentrations higher than 0.5 m THF. Attempts to telescope this final demethylation with the previous three‐step sequence in Scheme 8 led to an 8 % isolated yield over four steps; however, the same clogging issue meant the system could not be run for more than 10 h at a time.

**Scheme 9:**
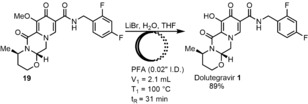
Demethylation to form DTG **1**.

In conclusion, we have developed a continuous flow synthesis of the HIV integrase inhibitor Dolutegravir **1**. The optimized process described involved seven total steps in three separate flow operations in 24 % overall yield (37 % overall when Step 3 was run as a separate flow operation).^19^ The key features of the flow route are rapid manufacturing time, direct amidation of ester **16** to reduce the step count, and separation of the acetal deprotection/oxazine formation flow reactors to attain high reactivity and selectivity for tricyclic product DTG‐OMe **19**. Importantly, our synthesis should be adaptable to both Cabotegravir **4** and Bictegravir **5** by switching the benzylamine and amino alcohol used in the synthesis. Further studies will focus on the telescoping of all steps to achieve an end‐to‐end continuous flow synthesis as well as formulation of the final API as its sodium salt and to produce cGMP formulations in an engineered system without the use of silica gel chromatography.

## Conflict of interest

The authors declare no conflict of interest.

## Supporting information

As a service to our authors and readers, this journal provides supporting information supplied by the authors. Such materials are peer reviewed and may be re‐organized for online delivery, but are not copy‐edited or typeset. Technical support issues arising from supporting information (other than missing files) should be addressed to the authors.

Supplementary
