# Supplementary material for: 7‐Step Flow Synthesis of the HIV Integrase Inhibitor Dolutegravir
Source: Angew Chem Int Ed Engl. 2018 May 14;57(24):7181–5. doi: 10.1002/anie.201802256 (PMC6033037; doi:10.1002/anie.201802256)
Supplement: Supplementary file 1 — Supplementary [file ANIE-57-7181-s001.pdf]

Angewandte Chemie
Eine Zeitschrift der Gesellschaft Deutscher Chemiker

Supporting Information

7-Step Flow Synthesis of the HIV Integrase Inhibitor Dolutegravir

Robert E. Ziegler, Bimbisar K. Desai, Jo-Ann Jee, B. Frank Gupton, Thomas D. Roper, and Timothy F. Jamison**

anie_201802256_sm_miscellaneous_information.pdf

Table of Contents

I. GENERAL CONSIDERATIONS……………………………………………...……....................S3
A. Methods...S3
B. Materials and Reagents...S3
C. Instrumentation...S3
II. EXPERIMENTAL PROCEDURES AND COMPOUND CHARACTERIZATION….................S5
A. General Procedures and Compound Characterization………………….………..........................S5
B. Selected Spectra…………………………………………………….……………….............S13
C. Pictures of Flow Setup...S18

1. General Considerations

Methods:

Stainless steel syringes were used to transfer air- and moisture-sensitive liquids. Reactions were monitored by thin-layer chromatography (TLC) on Silica Gel 60 F_{254} plates (EMD) under UV light (254 nm) or visualized with $KMnO_4$ upon heating. Flash chromatography was performed using Silica Gel 60 (230-400 mesh, EM Science), SiliaFlash P60 (230-400 mesh, SiliCycle), or 200-400 mesh silica gel (Sorbent Technologies). Organic solutions were concentrated under reduced pressure on a Büchi or Heidolph rotary evaporator. Analytical thin-layer chromatography (TLC) was accomplished with UV light (254 nm), and column chromatography was mediated on a Biotage Isolera flash chromatography system using SNAP KP-Sil or Ultra-Sil columns (silica gel, average particle size 50 µM).

Continuous flow equipment was assembled from commercially available components supplied from IDEX Health & Science Technologies, and backpressure regulators were purchased from Zaiput Flow Technologies. Reactors were constructed from high-purity perfluoroalkoxy (PFA) tubing with a 1/16" outside diameter and either 0.02″ or 0.04″ inside diameter as well as stainless steel (SS) tubing with a 1/16" outside diameter and 0.04″ inside diameter, with complementary PEEK fittings. Harvard Apparatus PhD Ultra syringe pumps were used to infuse solutions contained within 8 mL high-pressure stainless steel syringes with 1/16" SWAGELOCK® fitting. The check valves used had a CV inlet 1/4-28 (Perfluor), T-mixers were IDEX Tee Assy 1/8" PEEK 0.050 through, and ferrules were SF, SS Ring, 1/16", TZ.

Materials and reagents:

Commercial reagents were purchased from Millipore Sigma, Alfa Aesar, Strem, Oakwood Chemical, Acros Organics, Fisher Scientific, Matrix Scientific, or TCI and used without purification unless otherwise indicated. Methyl-4-methoxyacetoacetate (97%) was distilled prior to use in the first step of the flow synthesis. Sodium methoxide solution in methanol was purchased from Alfa Aesar and used as received. Where specified, CH_3CN, CH_2Cl_2, toluene, THF, and 1,4-dioxane (PURE SOLV) were dried by passing through columns of activated alumina. Deuterated solvents $CDCl_3$, CD_3OD, and DMSO-d_6 (Cambridge Isotope Laboratories or Millipore Sigma) were used without purification. Extraction and chromatography solvents were reagent grade and used without purification (VWR, Fisher Scientific or Millipore Sigma). Molecular sieves (4Å) were activated by heating *in vacuo* and stored in a vial in a dessicator. HPLC solvents were used without purification (VWR, Fisher Scientific).

Instrumentation:

Proton nuclear magnetic resonance (^1H NMR) spectra and proton-decoupled carbon nuclear magnetic resonance ($^{13}C\{^1H\}$ NMR) spectra were recorded on either a Bruker 400 MHz, Bruker 600 MHz, Varian 300 MHz, or Varian Inova 500 MHz NMR instrument spectrometer at ambient temperature. All chemical shifts (δ) are reported in parts per million (ppm) downfield from tetramethylsilane. Proton resonances are referenced to residual protium in the NMR solvent or tetramethylsilane. Carbon resonances are referenced to the carbon resonances of the NMR solvent.

Data are represented as follows: chemical shift, multiplicity (br = broad, s = singlet, d = doublet, t = triplet, q = quartet, m = multiplet, o = overlap), coupling constants (J) in Hertz (Hz), integration.

High-resolution mass spectrometry data were acquired by the Department of Chemistry Instrumentation Facility, Massachusetts Institute of Technology on a Bruker Daltonics APEXIV 4.7 Tesla FT-ICR Mass Spectrometer. Gas chromatography (GC) was performed on an Agilent 5870 GC (HP-5 column) with a flame ionization detector. GC/MS was performed on an Agilent 5870 GC (HP-5ms column) with an Agilent 5975C MSD. High-performance liquid chromatography (HPLC) analysis was performed using an Agilent 1200 series quaternary HPLC system with commercially available ChiralPak and ChiralCel columns. Liquid Chromatography Mass Spectrometry (LCMS) was recorded on an Agilent 6135 single quad (LCMS-ESI) with Phenomenex Gemini C18 110Å LC column (50 x 3 mm) at Virginia Commonwealth University. LCMS method is listed in Appendix 1.

Abbreviations used:

aq = aqueous, c = concentration (in grams/100 mL), calc'd = calculated, cm^{-1} = wavenumber, equiv = equivalents, h = hours, min = minutes, *m/z* = mass to charge ratio, R_f = retention factor, rt = room temperature, sat'd = saturated, t_R = retention time, residence time (continuous flow), O.D. = outside diameter, I.D. = inside diameter, v/v = volume/volume. CH_3CN = acetonitrile, AADMA = aminoacetaldehyde dimethylacetal, DMF = *N*,*N*-dimethylformamide, DMSO = dimethylsulfoxide, EtOAc = ethylacetate, M4MAA= methyl-4-methoxyacetoacetate, DMF-DMA = dimethylformamide dimethylacetal, HMDS = hexamethyldisilazide = bis(trimethylsilyl)amide, LiOMe = lithium methoxide, MeOH = methanol, NaOMe = sodium methoxde, THF = tetrahydrofuran.

2. Experimental Procedures and Compound Characterization

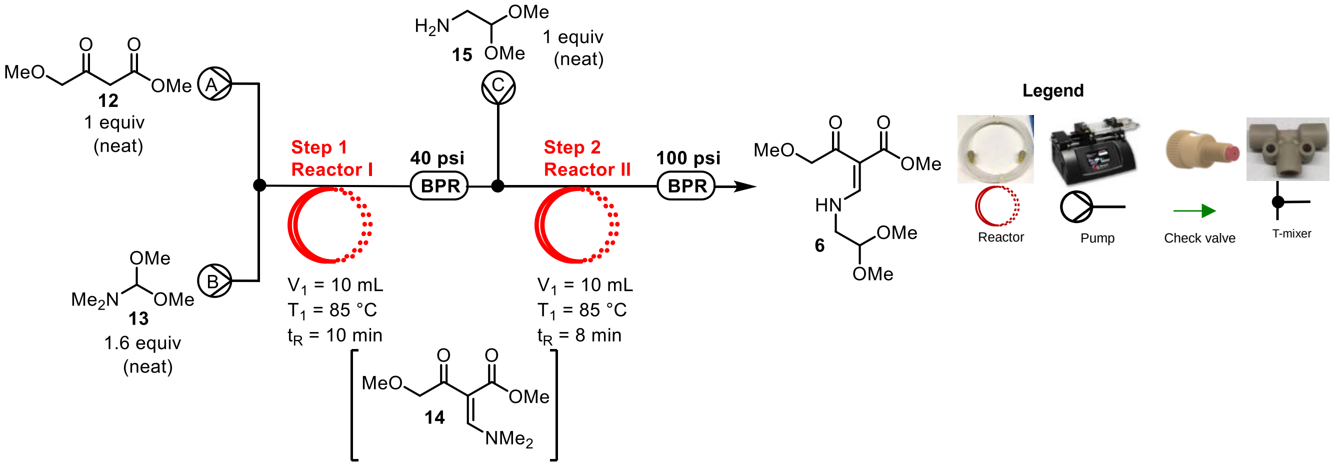

(*E*)-Methyl 2-(((2,2-dimethoxyethyl)amino)methylene)-4-methoxy-3-oxobutanoate 6: The Vapourtec R series flow reactor equipment consisting of two Knauer type HPLC pumps for reagent/solvent delivery and 2 x 10 mL PFA reactors (1/16" O.D. x 0.04" I.D.) was utilized in the flow chemistry experiment. Before the start of the actual experiment, all reactors were primed with $PhCH_3$. Reactor I was pre-heated to 85 °C and Reactor II was pre-heated to 85 °C. The Vapourtec Pump A was used to pump the neat stock of methyl-4-methoxyacetoacetate (M4MAA) **12** (50 mL, 1 equiv) and Vapourtec Pump B was used to pump the neat stock of dimethyl formamide dimethylacetal (DMF-DMA) **13** (60 ml, 1.6 equiv). Solutions A and B were mixed at a T-piece (M1) (IDEX Health and Science, P-726 used with blanking plugs) and pumped through Reactor I. A 40 psi back pressure regulator (BPR) (IDEX Health and Science, P-785) was connected after Reactor I (Step 1). The output from Reactor I was connected to a second T-piece (M2) with an incoming neat solution of aminoacetaldehyde dimethylacetal (AADMA) **15** (60 ml, 1 equiv) that was pumped using a Syrris ASIA Pump C. The collective flow stream was allowed to pump into Reactor II. A 100 psi back pressure regulator (BPR) (IDEX Health and Science, P-787) was connected after Reactor II. After approximately three total system residence times, the output flow from Reactor II was collected for 15 mins (19.8 mL, 32.9 mmol of rate limiting material). The reactant mixture was concentrated *in vacuo* and purified using flash chromatography (50-100% EtOAc/hexanes) to give pure **6** (7.16 g, 27.4 mmol, 95%).

Characterization Data: Off-white solid. R_f = 0.42 (100% EtOAc). ^{1}H NMR (600 MHz, $CDCl_3$) δ 10.98 (br. s, 1H), 7.96 (d, J = 13.8 Hz, 1H), 4.59 (s, 2H), 4.43 (m, 1H), 3.73 (s, 3H), 3.48 (s, 3H), 3.44 (o, 8H); ^{13}C NMR (125 MHz, $CDCl_3$) δ 196.9, 166.9, 160.9, 102.8, 98.5, 76.5, 59.1, 54.8, 51.9, 50.8.

Table S1. Solution concentration and flow rates for the synthesis of **6**.

Solution	Pump	Equiv	Concentration (M)	Flow rate (mL/min)
A	Vapourtec Pump A	1.0 equiv (**12**)	7.7	0.378
B	Vapourtec Pump B	1.6 equiv (**13**)	7.5	0.622
C	Syrris ASIA Pump C	1.5 equiv (**15**)	9.2	0.480

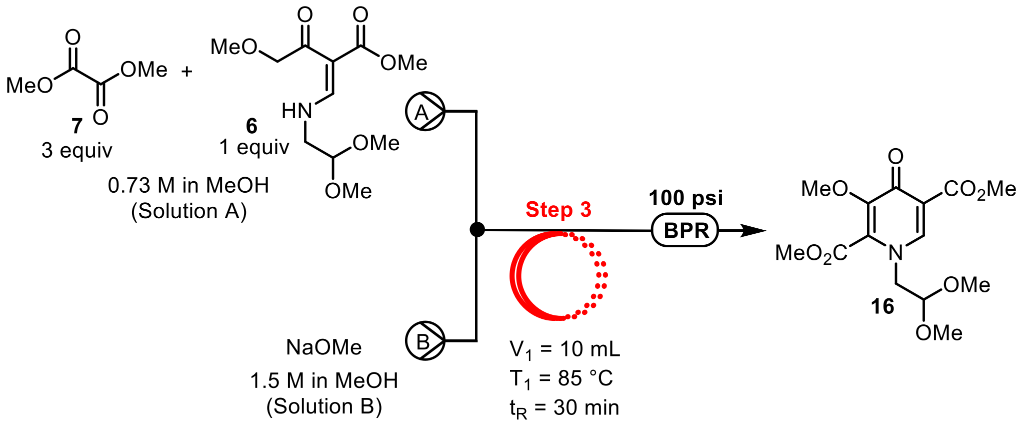

Dimethyl 1-(2,2-dimethoxyethyl)-3-methoxy-4-oxo-1,4-dihydropyridine-2,5-dicarboxylate 16: The Vapourtec R series flow reactor equipment consisting of two Knauer type HPLC pumps for reagent/solvent delivery and a 10 mL PFA reactor (1/16” O.D. x 0.04” I.D.) was utilized in the flow chemistry experiment. Before the start of the actual experiment, the reactor was primed with HPLC grade methanol.

Solution A: Vinylogous amide **6** (12 g, 45.9 mmol, 1 equiv) and dimethyl oxalate **7** (10.9 g, 91.8 mmol, 2 equiv) were dissolved in MeOH to afford a 63 mL solution (0.73 M)

Solution B: 20 mL of 25 wt% NaOMe solution in MeOH was dissolved in 40 mL MeOH to afford a 60 mL solution (1.5 M)

The reactor was then pre-heated to 85 °C. The Vapourtec Pump A was used to pump the stock solution A and Vapourtec Pump B was used to pump the stock solution B. Solutions A and B were mixed at the T-piece (M1) (IDEX, P-726 used with blanking plugs) and pumped through the reactor. A 100 psi back pressure regulator (BPR) (IDEX, P-785) was connected after the reactor. After approximately three total system residence times, the output flow from the reactor was collected for 60 mins (19.8 mL, 7.2 mmol of rate limiting material). The reactant mixture was concentrated *in vacuo* and the residue was then dissolved in 1 M citric acid solution. The aqueous phase was extracted with CH_2Cl_2 (3 x 15 mL) and the combined organic phase dried over anhydrous sodium sulfate. The solution was filtered under gravity and the filtrate was evaporated to dryness under reduced pressure on a rotary evaporator to afford pure **16** (2.2 g, 6.68 mmol, 91%).

Table S2. Solvent screen for the synthesis of compound **16**.

No.	Solvent	Base	20 min±	Solubility
1	MeOH	NaOMe 25 wt% in MeOH	85.9%	Soluble
2	NMP	NaOMe 25 wt% in MeOH	85.0%	Soluble
3	CH_3CN	NaOMe 25 wt% in MeOH	92.9%	Precipitated
4	MeOH	LiOMe 2.2M in MeOH	46.5%	Soluble
5	NMP	LiOMe 2.2M in MeOH	45.1%	Soluble
6	CH_3CN	LiOMe 2.2M in MeOH	43.0%	Cloudy

± % Conversion to product by HPLC (at 254 nM) response. Reaction mixture consisted of 3 equiv of dimethyl oxalate **7**, 1.5 equiv of base, and was run at 60 °C. Screening of other solvents such as toluene and diglyme with a combination of bases such as LDA, LiHMDS, KO*t*Bu, and Li_2O resulted in precipitation.

Table S3. Solution concentration and flow rates for the synthesis of **16**.

Solution	Pump	Equiv.	Concentration (M)	Flow rate (mL/min)
A	Vapourtec Pump A	1.0 equiv (**6**)<br>2.0 equiv (**7**)	0.73	0.165
B	Vapourtec Pump B	1.5 equiv	1.5	0.165

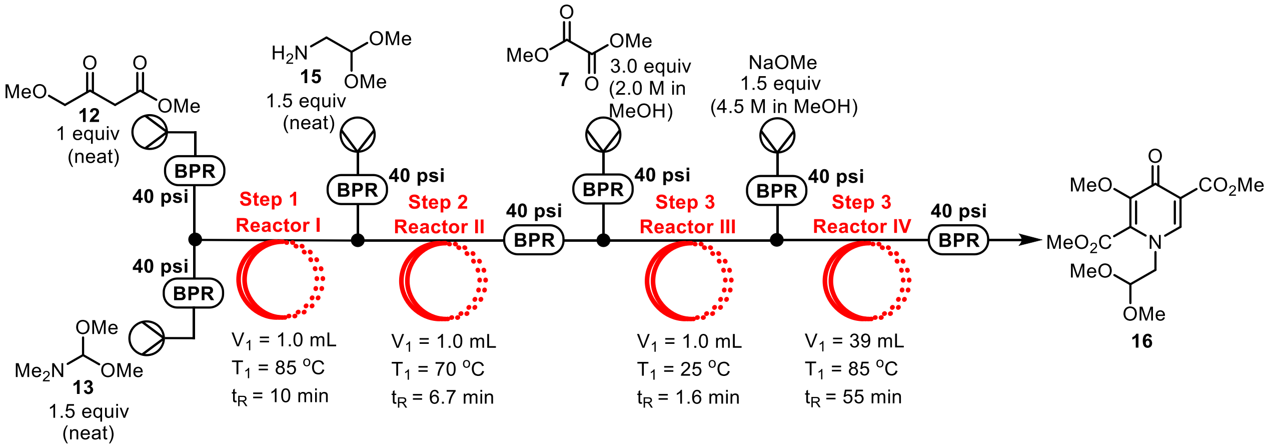

Dimethyl 1-(2,2-dimethoxyethyl)-3-methoxy-4-oxo-1,4-dihydropyridine-2,5-dicarboxylate 16: The three-step telescoped sequence was conducted in a continuous flow system without isolation of any intermediates or the in-line separation of any co-products, byproducts, or excess reagents. Methyl-4-methoxyacetoacetate **12** (10 mL, 1 equiv) and *N*,*N*-dimethylformamide dimethyl acetal **13** (10 mL, 1.5 equiv) were streamed neat at 40 µL/min and 60 µL/min, respectively, *via* a Chemyx Nexus 3000 infuse/withdraw syringe pump and pumped into a 1 mL ETFE tube reactor (0.02" I.D.) at 85 °C with a residence time of 10 min. The reactor outcome was streamed into a T-piece with aminoacetaldehyde dimethyl acetal **15** (10 mL, 1.5 equiv) at 50 µL/min (Chemyx Nexus 3000) and pumped into Reactor II, a 1 mL ETFE tube reactor (0.02" I.D.) for 6.7 min of residence time at 70 °C. The combined reactor outcome was streamed into a T-piece with a solution of preheated dimethyl oxalate **7** (20.0 g, 3.0 equiv, 2 M) in methanol at 40 °C at 0.46 mL/min (Eldex Optos series high pressure liquid metering pump) and pumped into Reactor III, a 1 mL ETFE tube reactor (0.04" I.D.) at 25 °C with a residence time of 1.6 min. The combined reactor outcome was then streamed into a T-piece with NaOMe 25% w/w in MeOH (20 mL, 1.5 equiv) at 0.10 mL/min (Eldex Optos series) into Reactor IV, a 39 mL ETFE tube reactor (0.04" I.D.) at 85 °C with a residence time of 55 min to yield an orange solution. At a total flow rate of 0.71 mL/min, the crude reaction mixture was collected for 25 mins. The reactant mixture was concentrated *in vacuo* and the residue was then dissolved in 11 mL of 1 M citric acid solution. The aqueous phase was extracted with CH_2Cl_2 (3 x 15 mL) and the combined organic phase dried over anhydrous sodium sulfate. The reactant mixture was concentrated *in vacuo* and purified using flash chromatography (50-100% EtOAc/hexanes) to give pure **16** (1.4 g, 4.25 mmol, 56%).

Characterization Data: Yellow solid. R_f = 0.16 (100% EtOAc). ^{1}H NMR (600 MHz, $CDCl_3$): δ 8.13 (s, 1H), 4.50 (t, *J* = 4.9 Hz, 1H), 3.98 (o, 6H), 3.92 (d, *J* = 4.86 Hz, 2H), 3.91 (s, 3H), 3.40 (s, 6H); ^{13}C (125 MHz, $CDCl_3$): δ 171.3, 165.8, 162.7, 150.7, 146.3, 133.9, 118.6, 103.0, 60.8, 56.9, 56.0, 53.6, 52.6.

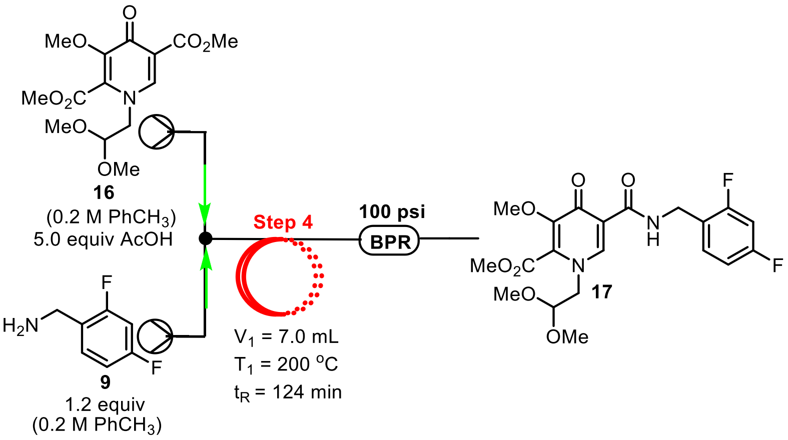

Methyl 5-((2,4-difluorobenzyl)carbamoyl)-1-(2,2-dimethoxyethyl)-3-methoxy-4-oxo-1,4-dihydropyridine-2-carboxylate 17: Flow setup was assembled with a 0.05" thru T-mixer (1/8" PEEK) at the entry to the reactor which consisted of stainless steel tubing 1/16" x 0.04" x 25'. Two solutions were prepared in 10 mL vials, and 7 mL of the resulting solutions were loaded into dry stainless steel Harvard syringes. Zaiput BPR was set at 100 psi, with the output directly going into a vial.

Solution 1: Ester **16** (1.05 g, 3.2 mmol, 1.0 equiv) and AcOH (0.91 mL, 15.9 mmol, 5 equiv) were dissolved with $PhCH_3$ to afford a 7 mL solution.

Solution 2: Difluorobenzylamine **9** (0.46 mL, 3.8 mmol, 1.2 equiv) was dissolved with $PhCH_3$ to afford a 7 mL solution.

Harvard syringe pumps were set to infuse at 25 μL min^{-1} (total flow rate = 50 μL min^{-1}), and the solution collected at the outlet of the continuous flow system for an equilibration period of 360 min. At this time, the reactant mixture was collected for 60 min (3.0 mL, 0.69 mmol of rate limiting material) into a vial; when 8 mL stainless steel syringes were empty, the syringes were recharged with 6 mL of dry $PhCH_3$ as a carrier solvent. The reactant mixture was concentrated *in vacuo* and purified using flash chromatography (10-80% EtOAc/hexanes) to give pure **17** (290 mg, 0.66 mmol, 96% yield).

Characterization Data: Yellow solid; R_f = 0.30 (3:2 EtOAc/hexanes). ^{1}H NMR (500 MHz, $CDCl_3$) δ 10.37 (br. s, *N*H), 8.40 (s, 1H), 7.36 (dd, *J* = 8.8, 6.9 Hz, 1H), 6.80 (o, 2H), 4.62 (d, *J* = 5.9 Hz, 2H), 4.49 (t, *J* = 4.9 Hz, 1H), 4.04 (m, 2H), 3.98 (s, 3H), 3.95 (s, 3H), 3.38 (s, 6H); ^{13}C NMR (125 MHz, $CDCl_3$) δ 173.3, 164.2, 162.5 (dd, *J* = 249, 12 Hz), 162.3, 160.9 (dd, *J* = 249, 12 Hz), 149.6, 144.6, 135.0, 130.9 (dd, *J* = 9.5, 9.5 Hz), 121.8 (dd, *J* = 15, 3.6 Hz), 119.5, 111.4 (dd, *J* = 21, 3.6 Hz), 103.9 (t, *J* = 25 Hz), 102.8, 60.9, 56.9, 55.8, 53.5, 52.1, 36.2 (d, *J* = 6.9 Hz).

Batch Optimization Screen

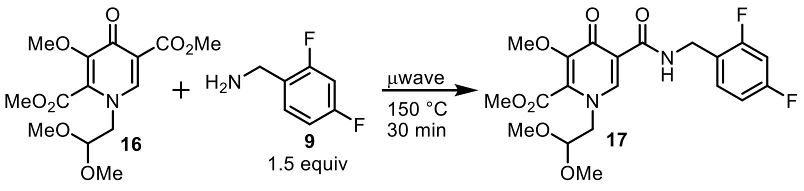

entry	solvent	acid	yield (%)	SM (%)
1	NMP	$Mg(OTf)_2$	Poor solubility	
2	CH_3CN	$B(OH)_3$	Poor solubility	
3	$PhCH_3$	$BF_3{\cdot}(OEt)_2$	68	14
4	$PhCH_3$	Ti(O*i*-Pr)$_4$	51	22
5	$PhCH_3$	TFA	50	30
6	$PhCH_3$	HCOOH	67	26
**7**	**$PhCH_3$**	**AcOH**	**83**	**11**

Flow Optimization

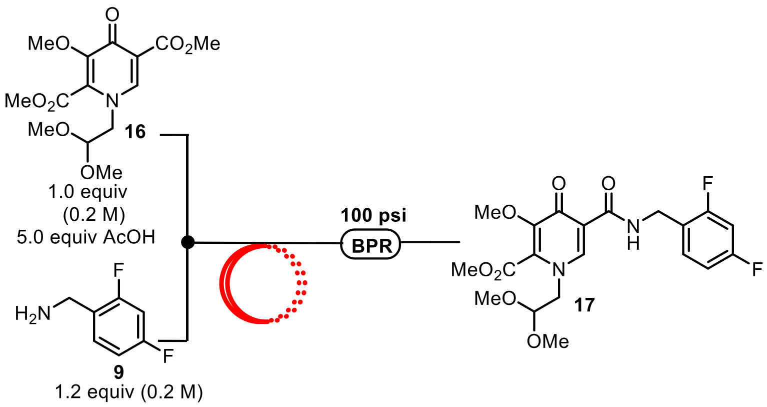

entry	solvent	temp (°C)	t_R (min)	yield (%)	SM (%)
1	$PhCH_3$	120	60	28	58
2	$PhCH_3$	150	60	65	27
3	$PhCH_3$	180	140	90	6
**4**	**$PhCH_3$**	**200**	**140**	**96**	**2**
5	Dioxane	150	60	58	32
6	CH_3CN	150	60	52	40

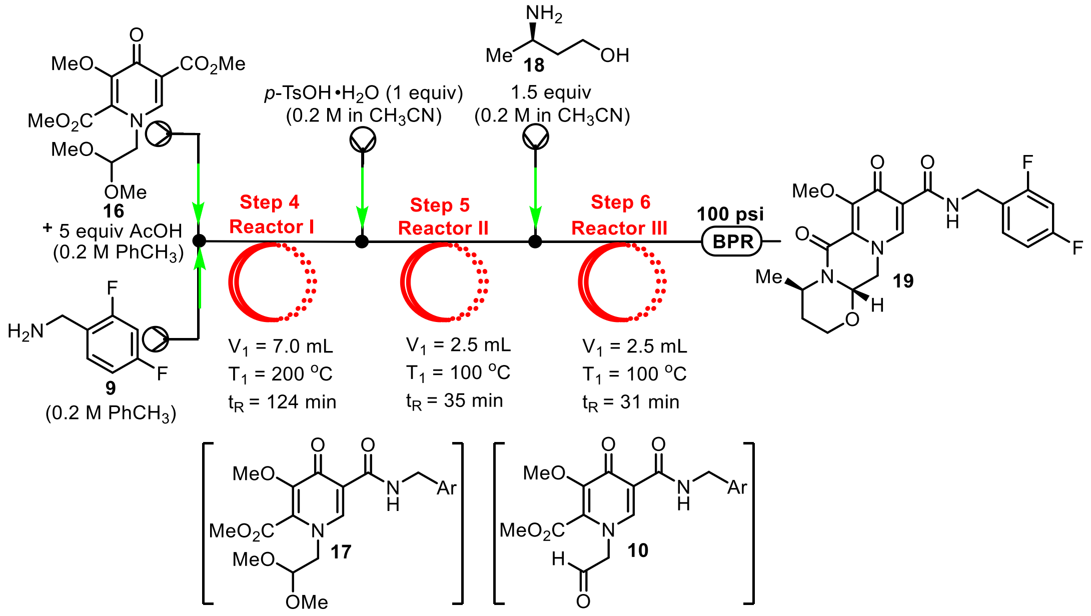

(4*R*,12a*S*)-*N*-(2,4-difluorobenzyl)-7-methoxy-4-methyl-6,8-dioxo-3,4,6,8,12,12a-hexahydro-2*H*-pyrido[1',2':4,5]pyrazino[2,1-b][1,3]oxazine-9-carboxamide 19: Flow setup was assembled with a 0.06” thru T-mixer at the entry to the reactor which consisted of stainless steel tubing 1/16” x 0.04” x 25'. The output of Reactor I was directly connected to a T-mixer into PFA tubing 1/16” x 0.04” x 10'. The output of Reactor II was directly connected to a T-mixer into PFA tubing 1/16” x 0.04” x 10'. Two solutions were prepared in 10 mL vials, and 6 mL of the resulting solutions were loaded into dry stainless steel Harvard syringes.

Solution 1: Ester **16** (1.07 g, 3.2 mmol, 1.0 equiv) and AcOH (0.92 mL, 15.9 mmol, 5 equiv) were dissolved with $PhCH_3$ to afford a 8 mL solution.

Solution 2: Difluorobenzylamine **9** (0.46 mL, 3.8 mmol, 1.2 equiv) was dissolved with $PhCH_3$ to afford a 8 mL solution.

Solution 3: *p*-Toluenesulfonic acid monohydrate (615 mg, 3.2 mmol, 1.0 equiv) was dissolved with CH_3CN to afford a 6 mL solution.

Solution 4: (*R*)-3-Aminobutan-1-ol **18** (0.47 mL, 4.9 mmol, 1.5 equiv) was dissolved with CH_3CN to afford a 6 mL solution.

Harvard syringe pump with solutions 1 and 2 was set to infuse at 25 μL min^{-1} (total flow rate in 1st reactor = 50 μL min^{-1}), Harvard syringe pump with solutions 3 and 4 was set to infuse at 20 μL min^{-1} (total flow rate in 2nd reactor = 70 μL min^{-1} , total flow rate in 3rd reactor = 90 μL min^{-1}) and the solution collected at the outlet of the continuous flow system for an equilibration period of 540 min. At this time, the reactant mixture was collected for 60 min (5.4 mL, 0.62 mmol of rate limiting material) into a vial with sat. $NaHCO_3$; when 8 mL stainless steel syringes were empty, the syringes were recharged with 6 mL of dry $PhCH_3$ as a carrier solvent and pumping was resumed. The aqueous layer was extracted with EtOAc and the reactant mixture was concentrated *in vacuo* and purified using flash chromatography (20-100% EtOAc/hexanes) to give pure **19** (131 mg, 0.32 mmol, 48% yield). Diastereomeric

ratio was calculated by integrating the crude ^{1}H NMR spectrum as well as HPLC analysis using ChiralPak AD-H column, which both gave a 7:1 ratio.

Characterization Data: White solid; R_f = 0.30 (100% EtOAc). ^{1}H NMR (500 MHz, $CDCl_3$) δ 10.36 (s, 1H), 8.37 (s, 1H), 7.35 (m, 1H), 6.8 (o, 2H), 5.21 (dd, *J* = 6.1, 3.8 Hz, 1H), 5.01 (m, 1H), 4.62 (d, *J* = 5.9 Hz, 2H), 4.25 (dd, *J* =13.3, 3.8 Hz, 1H), 4.12 (o, 1H), 4.03 (s, 3H), 3.97 (o, 2H), 2.2 (ddd, *J* = 8.6, 5.7, 5.7 Hz, 1H), 1.54 (dd, *J* = 13.3, 2.3 Hz, 1H), 1.36 (d, *J* = 7.0 Hz, 3H); ^{13}C NMR (125 MHz, $CDCl_3$) δ 175.0, 164.3, 163.3, 161.6 (dd, *J* = 249, 12 Hz), 159.8, 156.1, 155.2, 142.4, 131.1, 129.5, 119.4, 111.7, 104.2, 76.5, 62.9, 61.6, 54.0, 45.0, 37.0, 29.8, 16.5. **HR-MS**: *m/z* Calcd. for $C_{21}H_{21}F_2N_3O_5$ $[M+H]^+$: 434.41, Found 434.15 (-2.6 ppm).

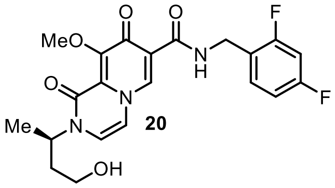

(*R*)-*N*-(2,4-difluorobenzyl)-2-(4-hydroxybutan-2-yl)-9-methoxy-1,8-dioxo-1,8-dihydro-2*H*-pyrido[1,2-a]pyrazine-7-carboxamide 20: The crude residue was purified by column chromatography (eluent: 1-10% MeOH/CH_2Cl_2) to give byproduct **20** (117 mg, 0.268 mmol, 88%). Yellow oil; R_f = 0.24 (100% EtOAc). ^{1}H NMR (500 MHz, $CDCl_3$) δ 10.56 (s, 1H), 8.61 (s, 1H), 7.37 (m, 1H), 6.81 (o, 2H), 6.72 (d, *J* = 6.3 Hz, 1H), 6.40 (d, *J* = 6.3 Hz, 1H), 5.08 (m, 1H), 4.65 (d, *J* = 5.9 Hz, 2H), 4.11 (o, 2H), 4.05 (s, 3H), 2.00 (o, H), 1.37 (d, *J* = 6.9 Hz, 3H); ^{13}C NMR (125 MHz, $CDCl_3$) δ 172.9, 170.8, 163.6, 162.1, 161.1, 159.5, 154.1, 153.8, 137.6, 130.7, 128.8, 121.1, 114.4, 111.4, 104.1, 61.0, 47.8, 36.8, 33.6, 20.9, 19.2. **HR-MS**: *m/z* Calcd. for $C_{21}H_{21}F_2N_3O_5$ $[M+H]^+$: 434.41, Found 434.15 (-2.6 ppm).

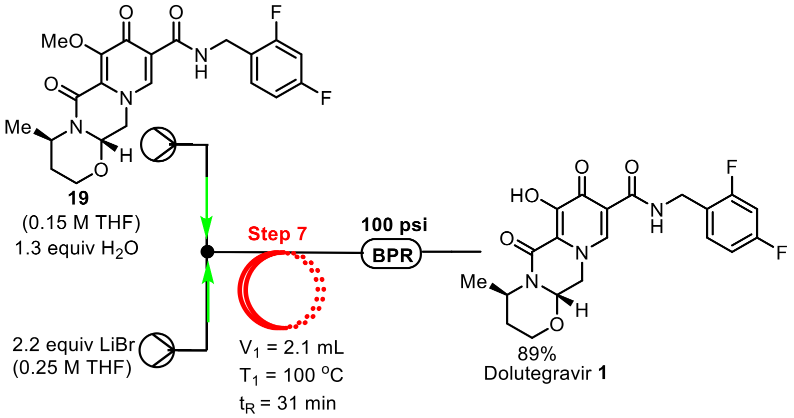

(4*R*,12a*S*)-*N*-(2,4-difluorobenzyl)-7-hydroxy-4-methyl-6,8-dioxo-3,4,6,8,12,12a-hexahydro-2*H*-pyrido[1',2':4,5]pyrazino[2,1-b][1,3]oxazine-9-carboxamide 1: Flow setup was assembled with a 0.06" thru T-mixer at the entry to the reactor which consisted of PFA tubing 1/16" x 0.02" x 20'. Two solutions were prepared in 10 mL vials, and 6 mL of the resulting solutions were loaded into dry stainless steel Harvard syringes.

Solution 1: DTG-OMe **19** (500 mg, 1.15 mmol, 1.0 equiv) and H_2O (30 µL, 1.5 mmol, 1.3 equiv) were dissolved with THF to afford a 8 mL solution.

Solution 2: LiBr (220 mg, 2.54 mmol, 2.2 equiv) was dissolved with THF to afford a 8 mL solution.

Harvard syringe pump was set to infuse at 20 µL min^{-1} (total flow rate in 1st reactor = 40 µL min^{-1}) and the solution collected at the outlet of the continuous flow system for an equilibration period of 90 min. At this time, the reactant mixture was collected for 30 min (1.2 mL, 0.09 mmol of rate limiting material) into a vial with 1M HCl. The aqueous layer was extracted with CH_2Cl_2 and the reactant mixture was concentrated *in vacuo* and purified as a solid by filtering after a slurry with THF/H_2O. The product could also be isolated by flash chromatography (1-20% MeOH/CH_2Cl_2) to give pure **19** (32 mg, 0.077 mmol, 89% yield).

Note: If concentration of the solutions is above 0.5 M, clogging was repeatedly experienced

Characterization Data: White solid; $\boldsymbol{R_f}$ = 0.28 (5% MeOH/CH_2Cl_2). ^{1}H NMR (500 MHz, $CDCl_3$) δ 12.43 (s, 1H), 10.36 (s, 1H), 8.31 (s, 1H), 7.34 (m, 1H), 6.78 (o, 2H), 5.25 (dd, *J* = 5.9, 4.0 Hz, 1H), 4.97 (ddd, *J* = 7.3, 6.0, 1.9 Hz, 1H), 4.62 (d, *J* = 5.9 Hz, 2H), 4.29 (dd, *J* = 13.5, 4.1 Hz, 1H), 4.12 (dd, *J* = 13.5, 6.0 Hz, 1H), 4.01 (o, 2H), 2.20 (m, 1H), 1.54 (dd, *J* = 14.0, 2.1 Hz, 1H), 1.39 (d, *J* = 7.0 Hz, 3H); ^{13}C NMR (125 MHz, $CDCl_3$) δ 171.4, 164.3, 163.3, 162.6, 161.8 (dd, *J* = 249, 12 Hz), 156.1, 140.3, 130.3, 121.7, 116.6, 115.9, 111.3, 103.8, 76.4, 62.8, 52.5, 44.8, 36.6, 29.4, 15.6.

Appendix 1a. LCMS method water (0.05% formic acid): methanol at a flow rate of 1 mL/min.

Time (min)	Water (%)	Methanol (%)
0.00	95.0	5.0
6.00	65.0	35.0
8.00	65.0	35.0
8.01	95.0	5.0

Appendix 1b. LCMS method water (0.05% formic acid): acetonitrile (0.05% formic acid) at a flow rate of 1 mL/min.

Time (min)	Water (%)	Acetonitrile (%)
0.00	95.0	5.0
1.00	95.0	5.0
6.00	5.0	95.0
8.00	5.0	95.0

3. Select Spectra

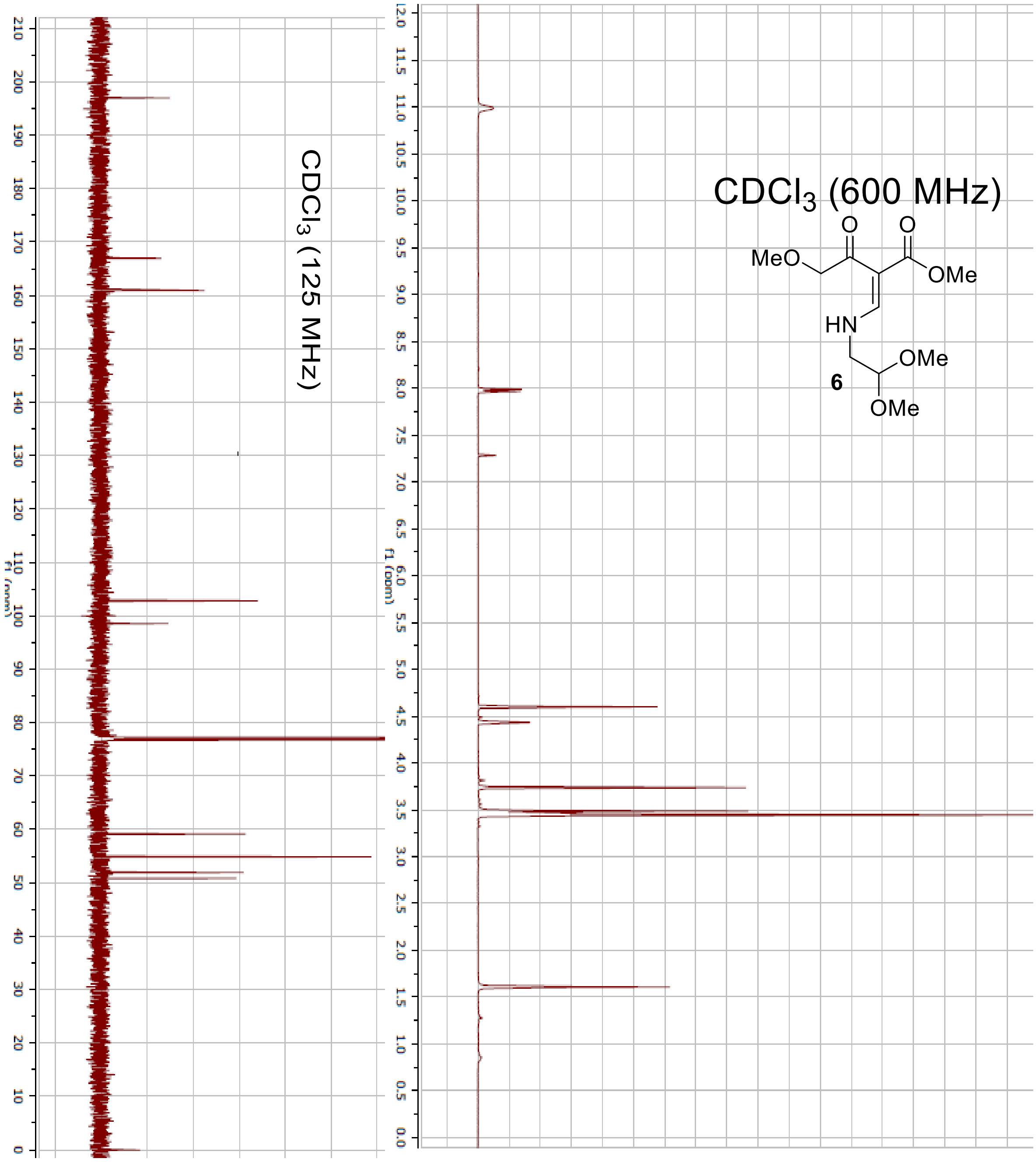

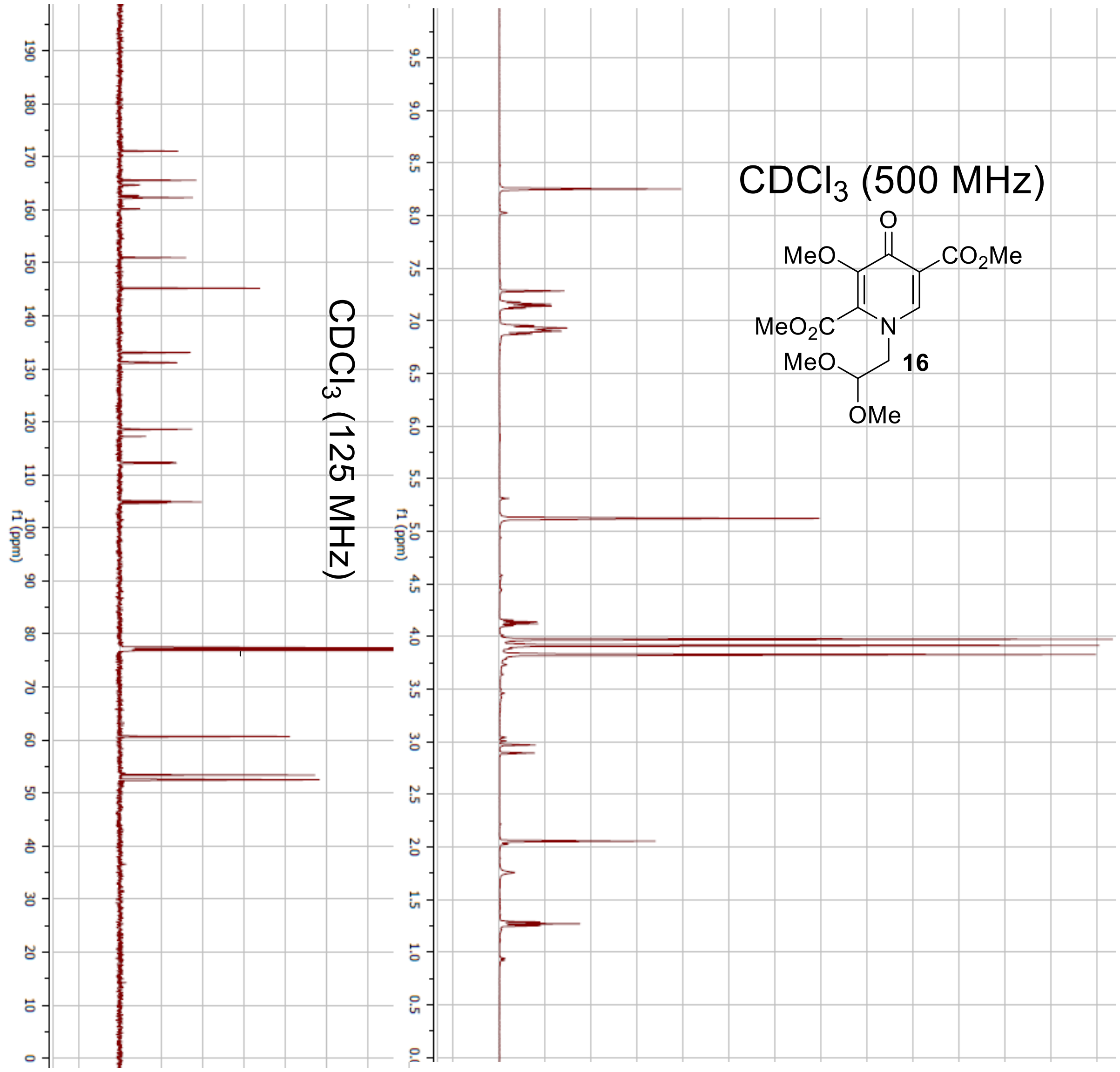
CDCl3 (500 MHz)
MeO
CO2Me
MeO2C
N
MeO
16
OMe
CDCl3 (125 MHz)
f1 (ppm)
f1 (ppm)

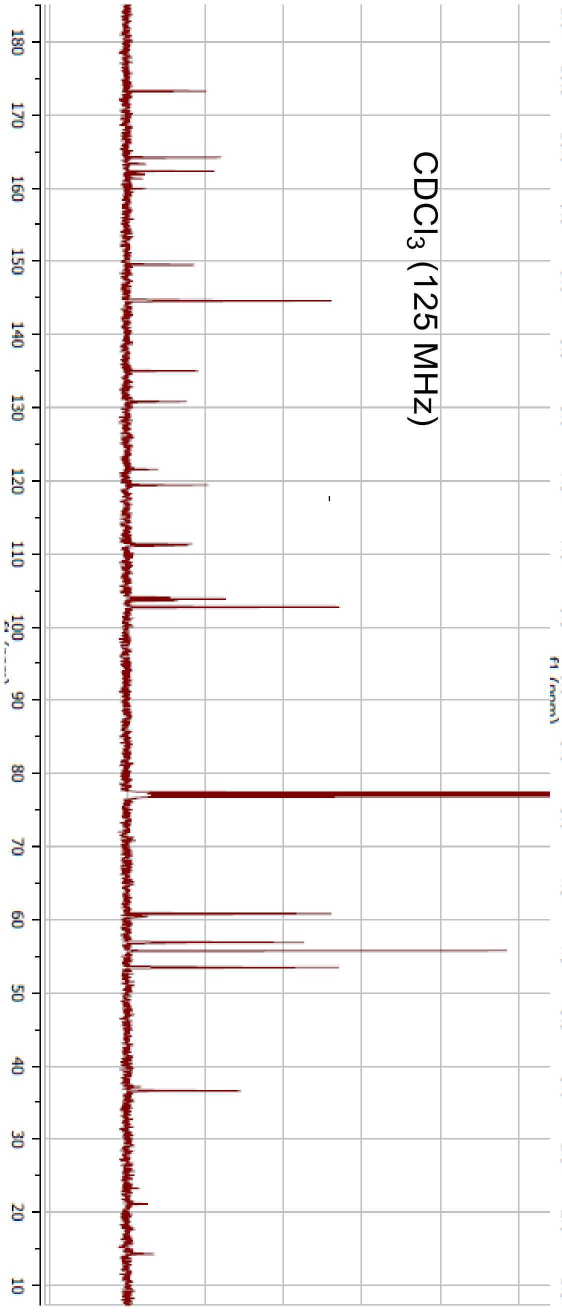
CDCl$_3$ (125 MHz)

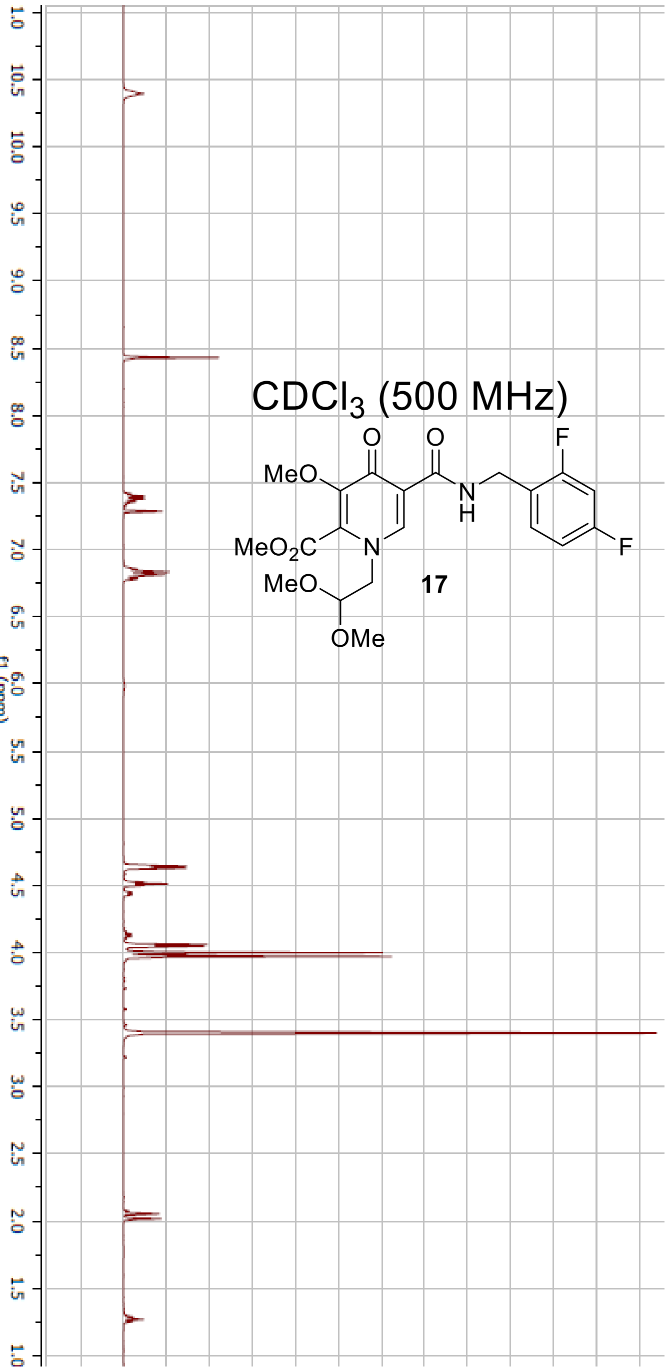
CDCl$_3$ (500 MHz)
MeO
MeO$_2$C
MeO
OMe
17

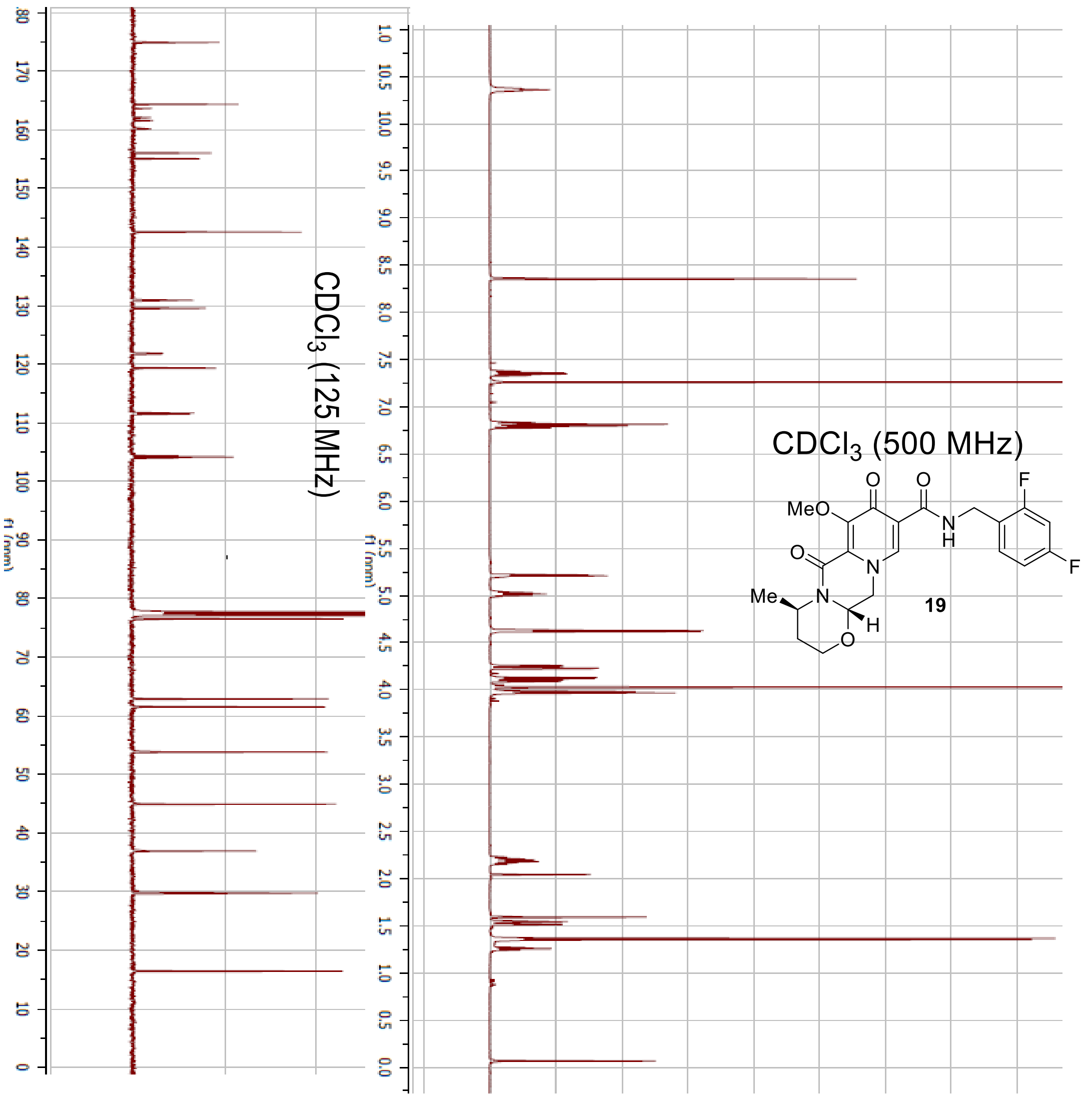
CDCl3 (125 MHz)
CDCl3 (500 MHz)
f1 (ppm)
MeO
Me
19
F

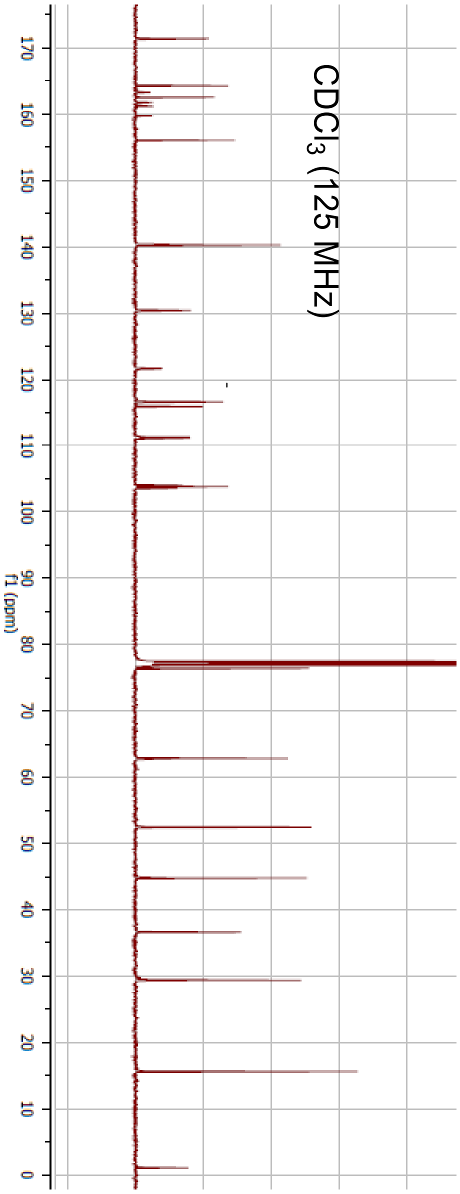
CDCl3 (125 MHz)
f1 (ppm)

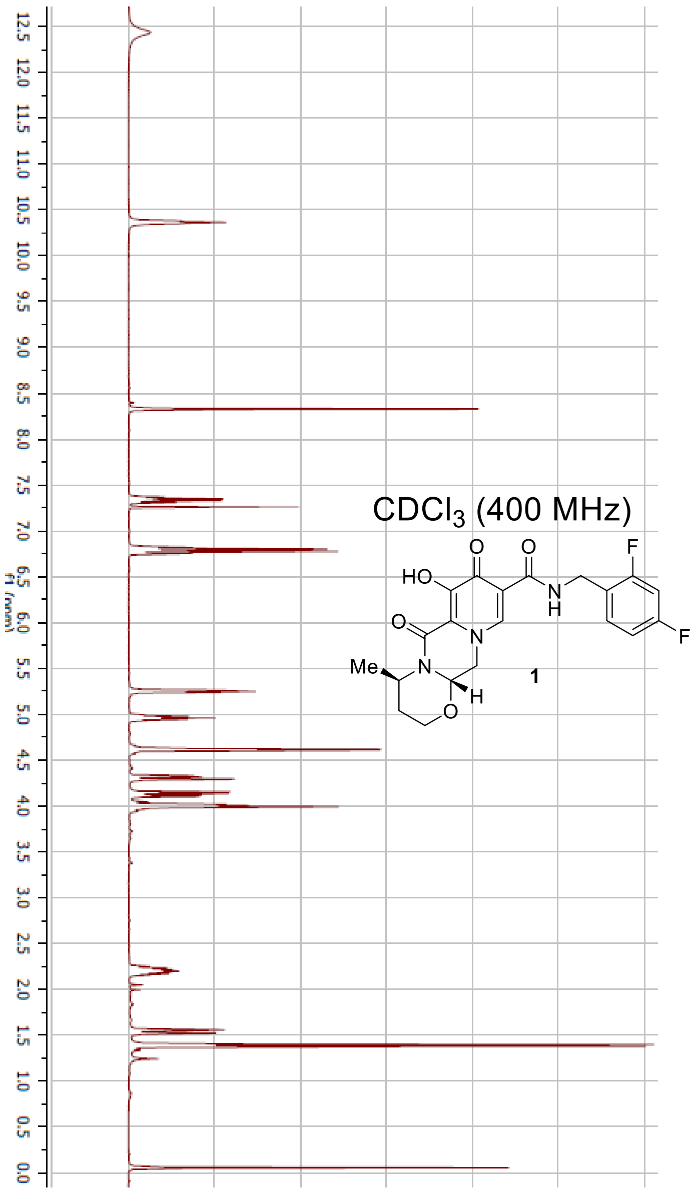
CDCl3 (400 MHz)
HO
O
O
N
H
F
F
O
N
Me
N
H
O
1
f1 (ppm)

4. Pictures of Flow Setup

Steps 1-3 Telescope

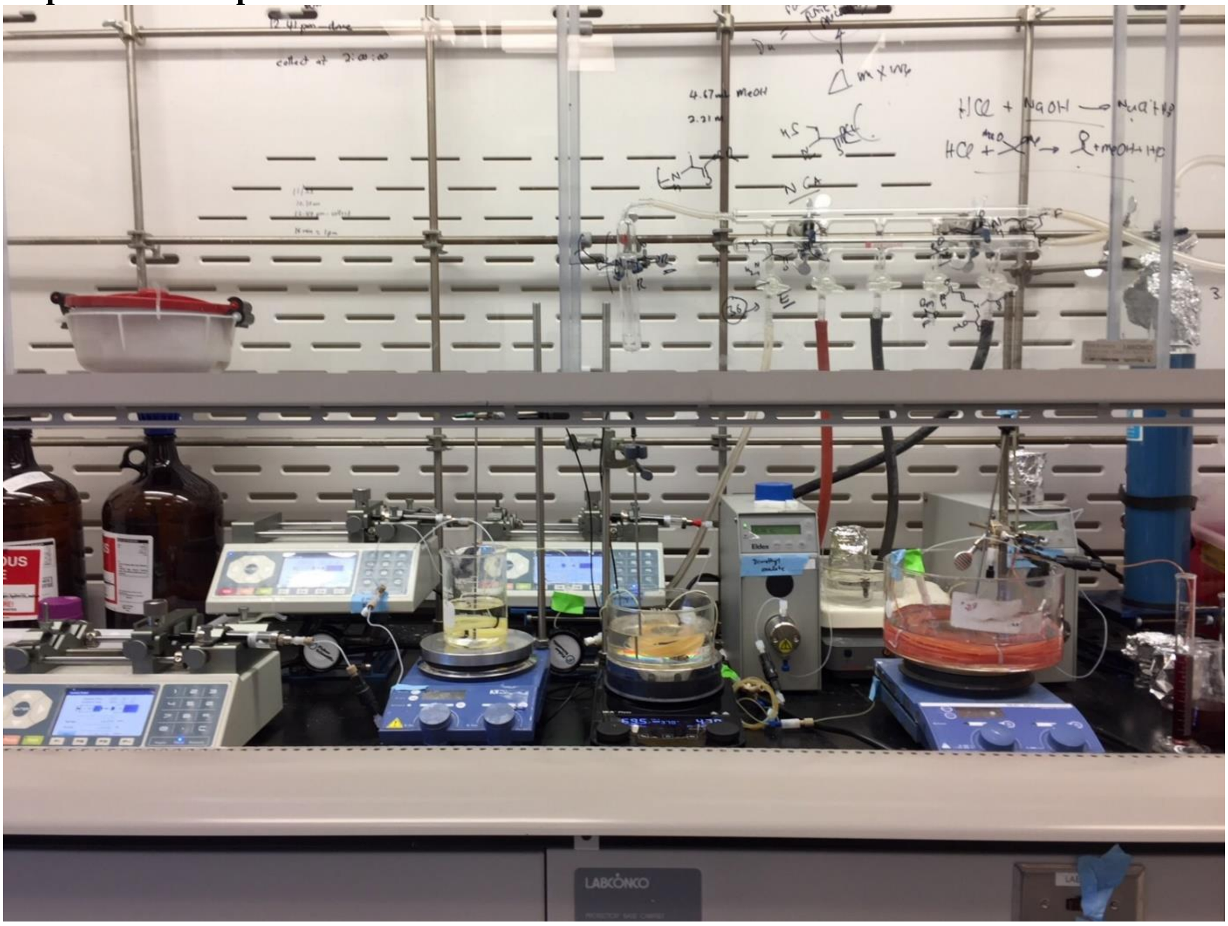

Step 4 Amidation

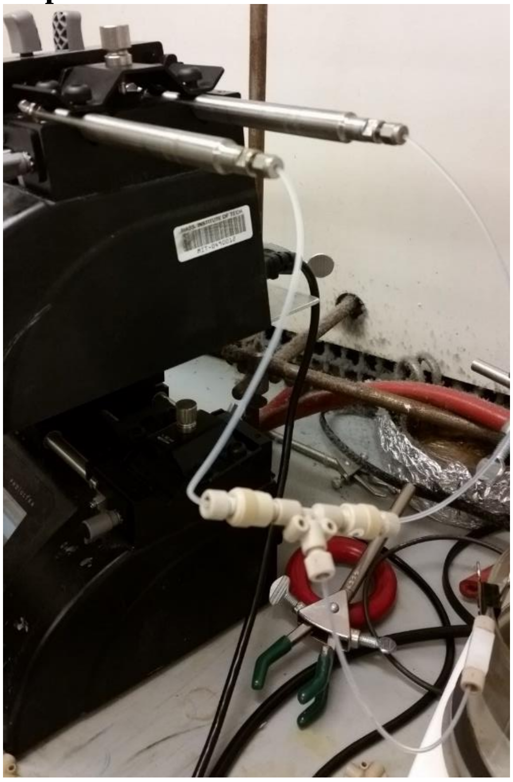

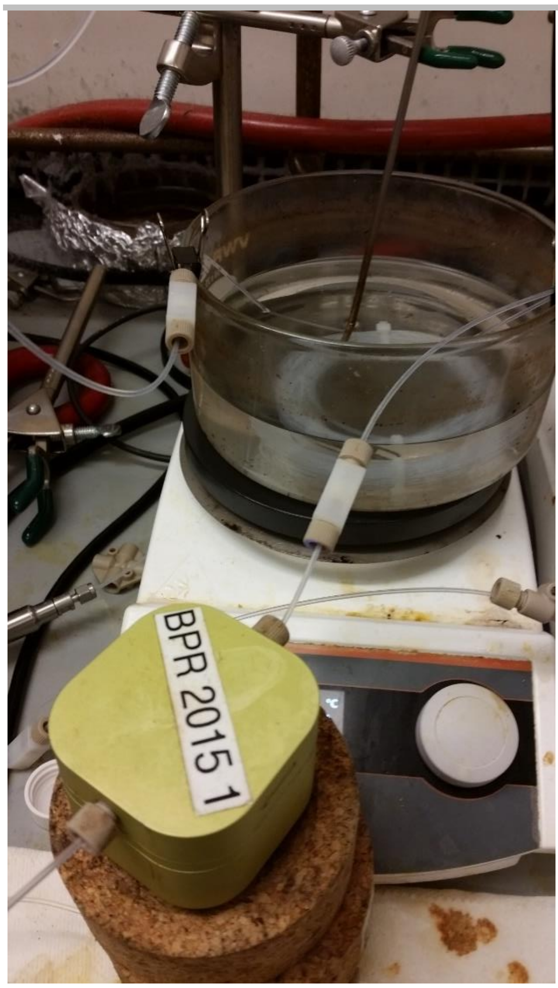
BPR 2015 1

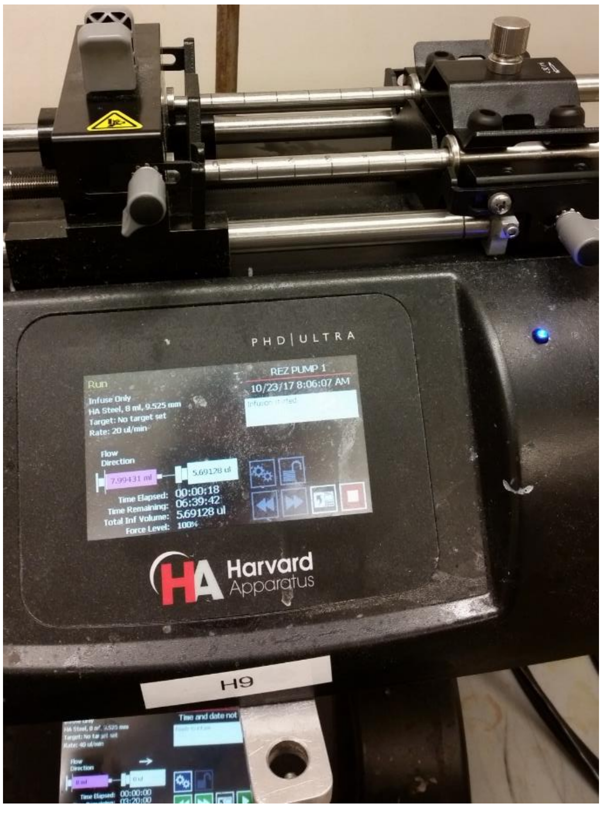
PHD | ULTRA
Run
REZ PUMP 1
10/23/17 8:06:07 AM
Infuse Only
HA Steel, 8 ml, 9.525 mm
Target: No target set
Rate: 20 ul/min
Flow Direction
Time Elapsed: 00:00:18
Time Remaining: 06:39:42
Total Inf Volume: 5.69128 ul
Force Level: 100%
Harvard Apparatus
H9

Steps 4-6 Telescoped Synthesis

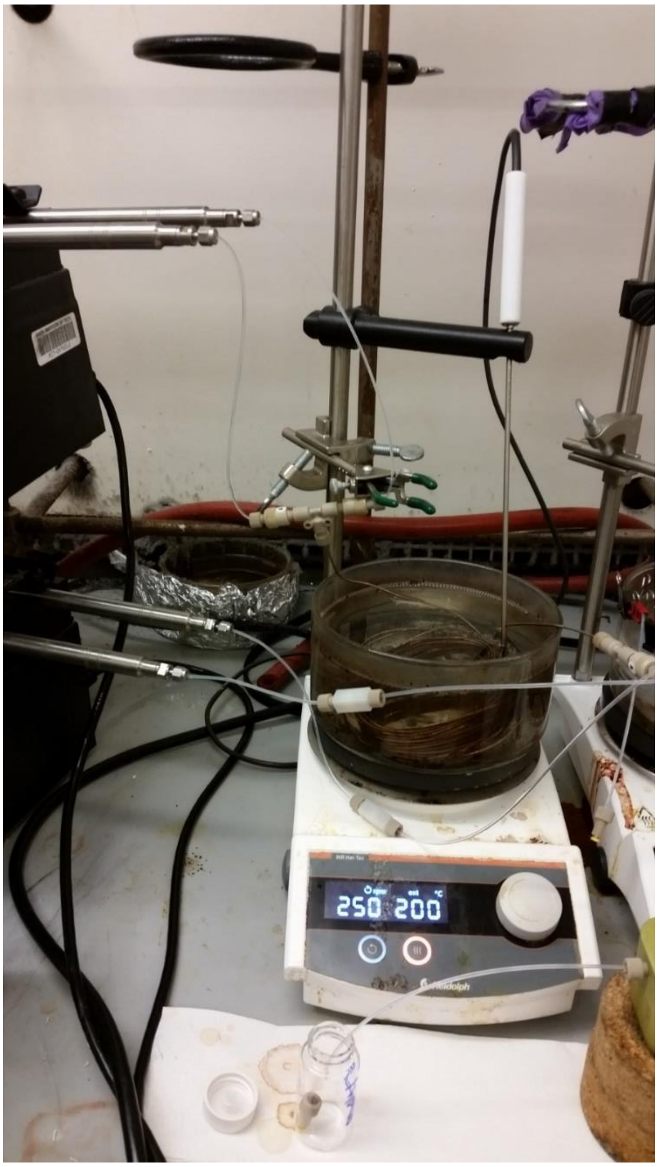

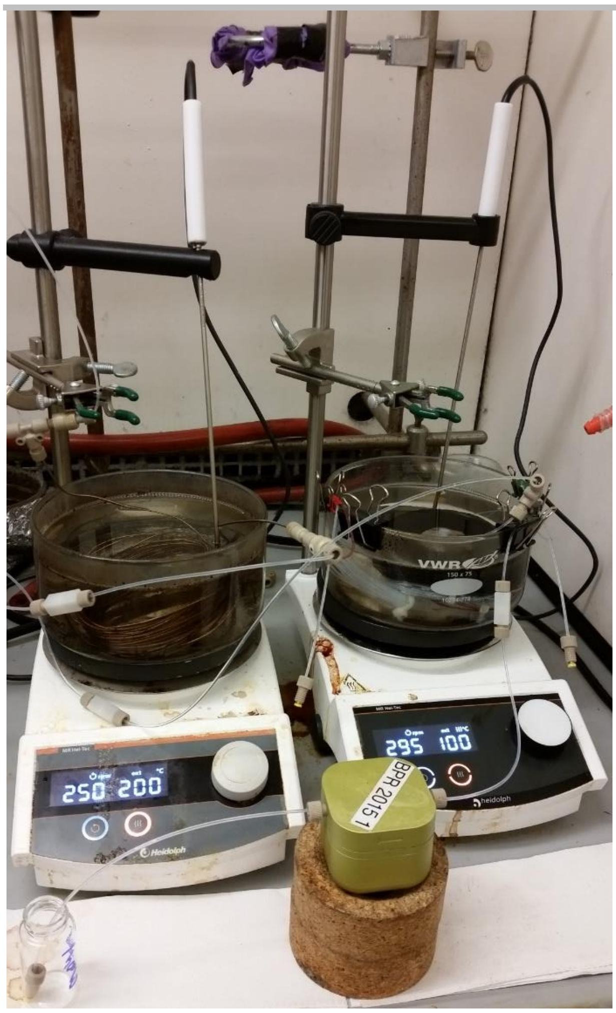
VWR
250 200
295 100
BPR 20151
Heidolph
heidolph

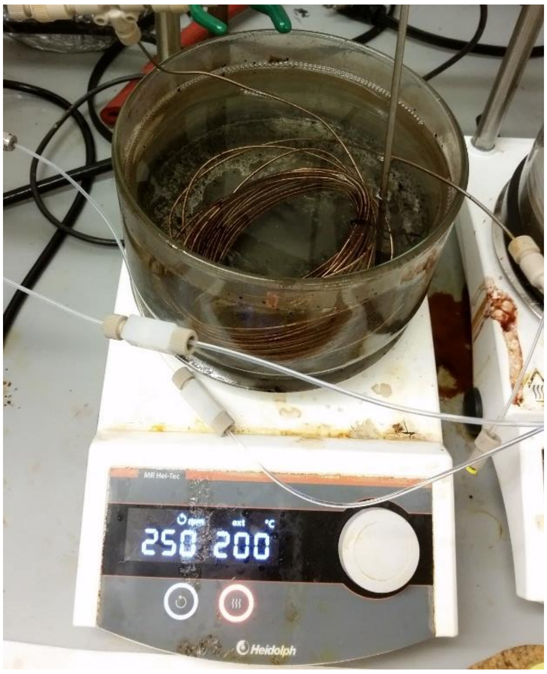
250 200
Heidolph

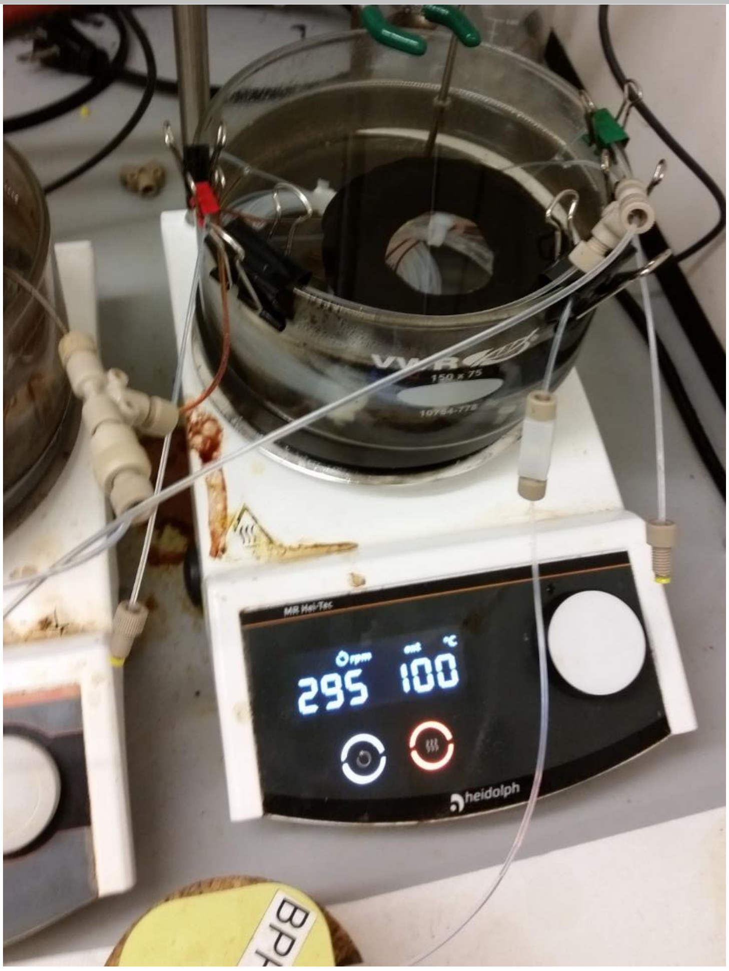
295
100
heidolph

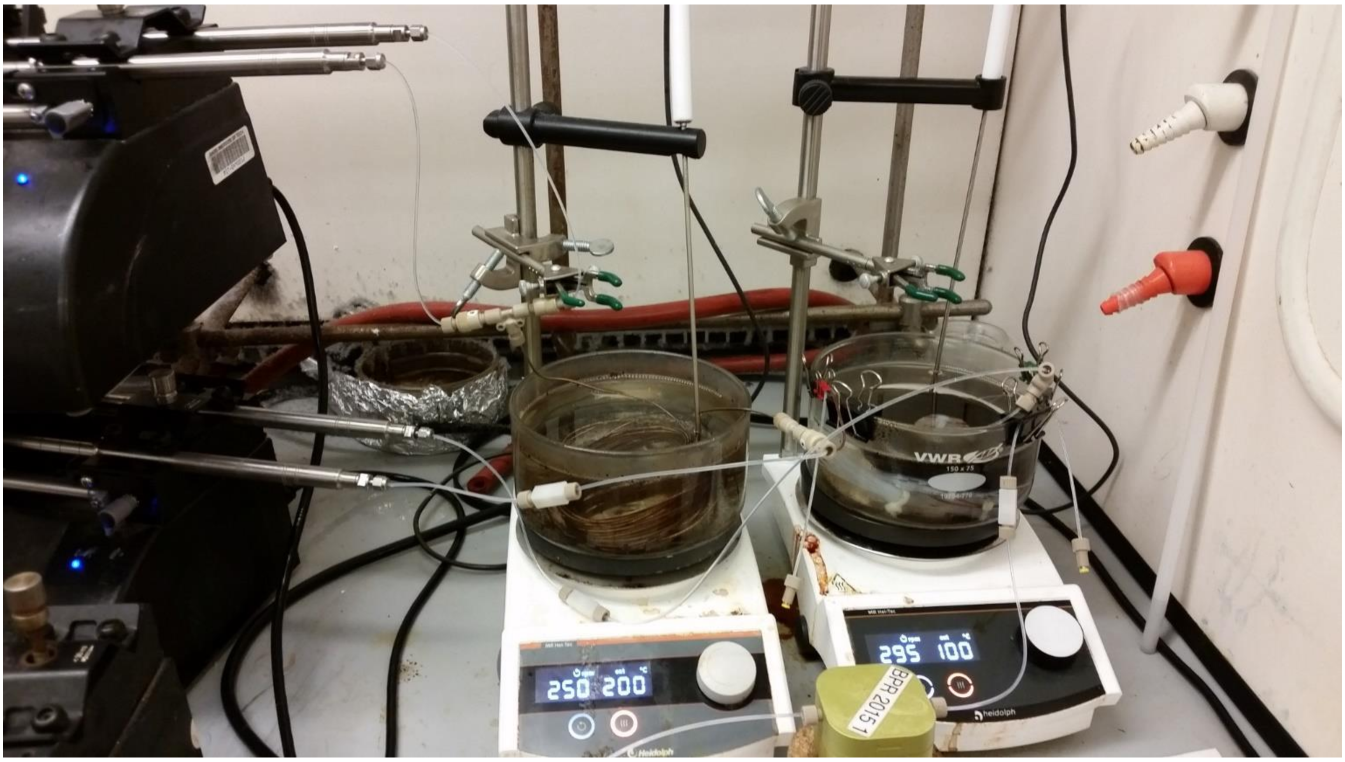
250
200
295
100
heidolph
